# Protecting *Pax6* 3′ UTR from MicroRNA-7 Partially Restores PAX6 in Islets from an Aniridia Mouse Model

**DOI:** 10.1016/j.omtn.2018.08.018

**Published:** 2018-09-01

**Authors:** Kevin Yongblah, Spencer C. Alford, Bridget C. Ryan, Robert L. Chow, Perry L. Howard

**Affiliations:** 1 Department of Biochemistry and Microbiology, University of Victoria, P.O. Box 1700 STN CSC, Victoria, BC V8W2Y2, Canada; 2 Department of Biology, University of Victoria, P.O. Box 1700 STN CSC, Victoria, BC V8W 2Y2, Canada

**Keywords:** aniridia, Pax6, microRNA, target protector, islets

## Abstract

Aniridia is a rare congenital syndrome that is associated with reduced visual acuity and progressive loss of vision. Aniridia patients may also develop systemic health issues associated with defects in the pancreas, digestive, and central nervous systems. The spectrum of symptoms associated with aniridia is due to haploinsufficiency of the paired box 6 gene (*PAX6*) and its role in the development and maintenance of the affected tissues. Here, we isolated pancreatic islets from mice heterozygous for *Pax6* to test whether a *Pax6*-specific miRNA suppression (target protector) strategy can restore PAX6 protein levels. We show that miR-7 and miR-375 target specific sites within the *Pax6* 3′ UTR in a mouse pancreatic β-insulinoma cell line. Tough decoys (Tuds) against miR-7 and miR-375 increase expression of a mouse *Pax6* 3′ UTR luciferase reporter and increase PAX6 protein levels in these cells. Finally, we demonstrate that the shielding of the miR-7 binding site with a target protector restores PAX6 protein levels in the *Pax6* heterozygous islets. The data presented here represent a proof of concept for RNA-based therapy for the progressive defects associated with aniridia and suggest the target protector approach may be a useful therapeutic strategy for other haploinsufficiency diseases.

## Graphical Abstract

**Figure:**
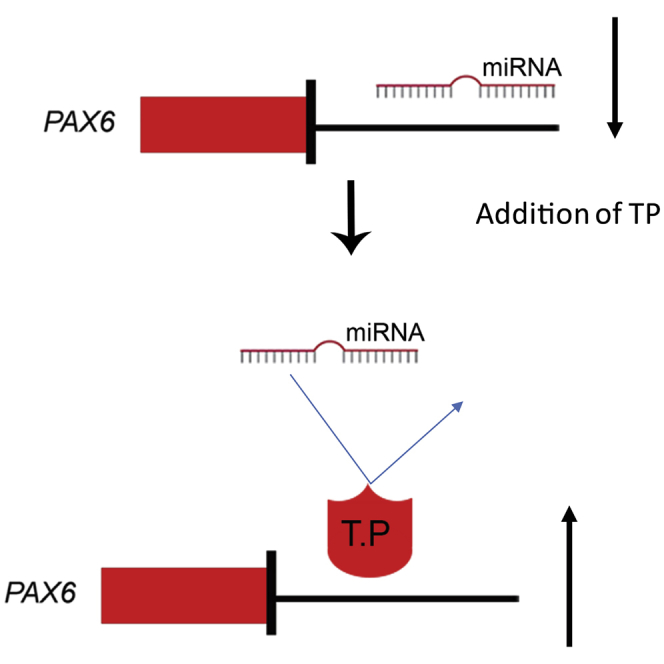

## Introduction

*PAX6* is a critical transcription factor for the development of the eye, brain, and pancreas. Haploinsufficiency of PAX6 leads to aniridia in humans, a rare eye disorder named for underdevelopment of the iris.^1^, ^2^ However, there is a spectrum of ocular symptoms associated with aniridia, and most major eye structures are impacted.^1^, ^2^ Patients are born with low vision primarily due to hypomorphic fovea but will frequently experience a progressive loss of vision due to cataracts, corneal clouding, and glaucoma.^1^ In addition, aniridia is associated with several non-ocular conditions such as obesity, glucose intolerance and diabetes, and anosmia.^3^, ^4^ These conditions are due to a requirement for *PAX6* expression for the development and maintenance of the brain, pancreas, and gut.^5^, ^6^, ^7^ The progressive nature of the disease reflects an ongoing requirement for *PAX6* expression and suggests there may be an opportunity for therapeutic intervention postnatally. Furthermore, haploinsufficiency associated with this disorder means that patients should be immune tolerant to PAX6 protein, making this disease a good candidate for gene therapy-based interventions.

A challenge for *PAX6* therapy is that PAX6 protein levels need to be tightly regulated: too much PAX6 can be as detrimental as too little. For example, overexpression in mice has been shown to lead to micropthalmia and pancreatic tumors.^8^, ^9^ In humans, a mutation within a microRNA-328 binding site within the 3′ UTR has been implicated in rolandic epilepsy.^10^ Similarly, PAX6 overexpression has been shown to promote pancreatic, breast, and non-small-cell lung cancer tumorigenesis.^9^, ^11^, ^12^, ^13^, ^14^ A further complication to gene therapy for aniridia is that there are two isoforms of PAX6, which differ in their sequence and function.^15^ Recently, a phase 2 clinical trial has begun for the treatment of aniridia patients using a topical formulation of the drug, Ataluren (ClinicalTrials.gov: NCT02647359).^16^ Ataluren targets the mutant allele by suppressing premature stop codons created by nonsense mutations.^17^ Therefore, its potential efficacy is limited to those patients with premature stop mutations where substitution with another amino acid does not disrupt the function of the protein. A priori prediction of which patients may benefit from the drug may be challenging and strategies are needed which treat all patients with this disorder.

Since most aniridic patients have one good copy of the *PAX6* gene, we reasoned that methods aimed at increasing the expression of the wild-type copy of the *PAX6* gene may be promising. Such approaches should benefit all patients regardless of the type of mutation, and since the *PAX6* gene is left under its endogenous chromatin environment, the inherent transcriptional regulation should be maintained. MicroRNA (miRNA) are small 21- to 22-bp non-coding RNA that regulate the expression of ∼60% of all genes.^18^, ^19^ Typically, miRNA bind to complementary sequences found within the 3′ UTR of their target mRNA and either suppress translation of the mRNA or trigger its degradation. A given gene is frequently regulated by several miRNA, such that each miRNA only weakly controls the protein output of the target.^18^ This potential for modest control exhibited by individual miRNA make them attractive targets for increasing expression of the wild-type copy of the *PAX6* gene. miR-7 and miR-375 are expressed in the pancreas and have been shown previously to regulate *PAX6.*^20^, ^21^, ^22^, ^23^, ^24^ Given the pancreatic involvement in aniridic patients, these miRNAs targeting *PAX6* provided an opportunity to address the question of whether targeting miRNA regulation of *PAX6* is feasible therapeutic strategy for aniridia.

In this report, we evaluate the effectiveness of using target protectors to block miR-7 and miR-375 sites within the 3′ UTR of the murine *Pax6* gene in a pancreatic cell line and islets isolated from mice heterozygous for the *Pax6*^*SeyDey+/*−^ mutation. Target protectors are small RNA designed to base-pair with miRNA sites within the 3′ UTR of the mRNA preventing the action of the miRNA on a specific target. We show that miR-7 and miR-375 provide a modest level of control over PAX6 protein levels in the beta cell insulinoma cell line (β-TC-6). In addition, a target protector targeting a miR-7 binding site within the 3′ UTR of *Pax6* effectively increases expression of PAX6 without resulting in overexpression in islets isolated from mice heterozygous for the *Pax6* ^*SeyDey+/*−^ mutation. Our results provide proof of concept for targeting of miRNA in treatment of aniridia and potentially other haploinsufficiency disorders.

## Results

### miR-7 and miR-375 Sites in the *Pax6* 3′ UTR Are Functional in Pancreatic Cells

**Figure 1:**
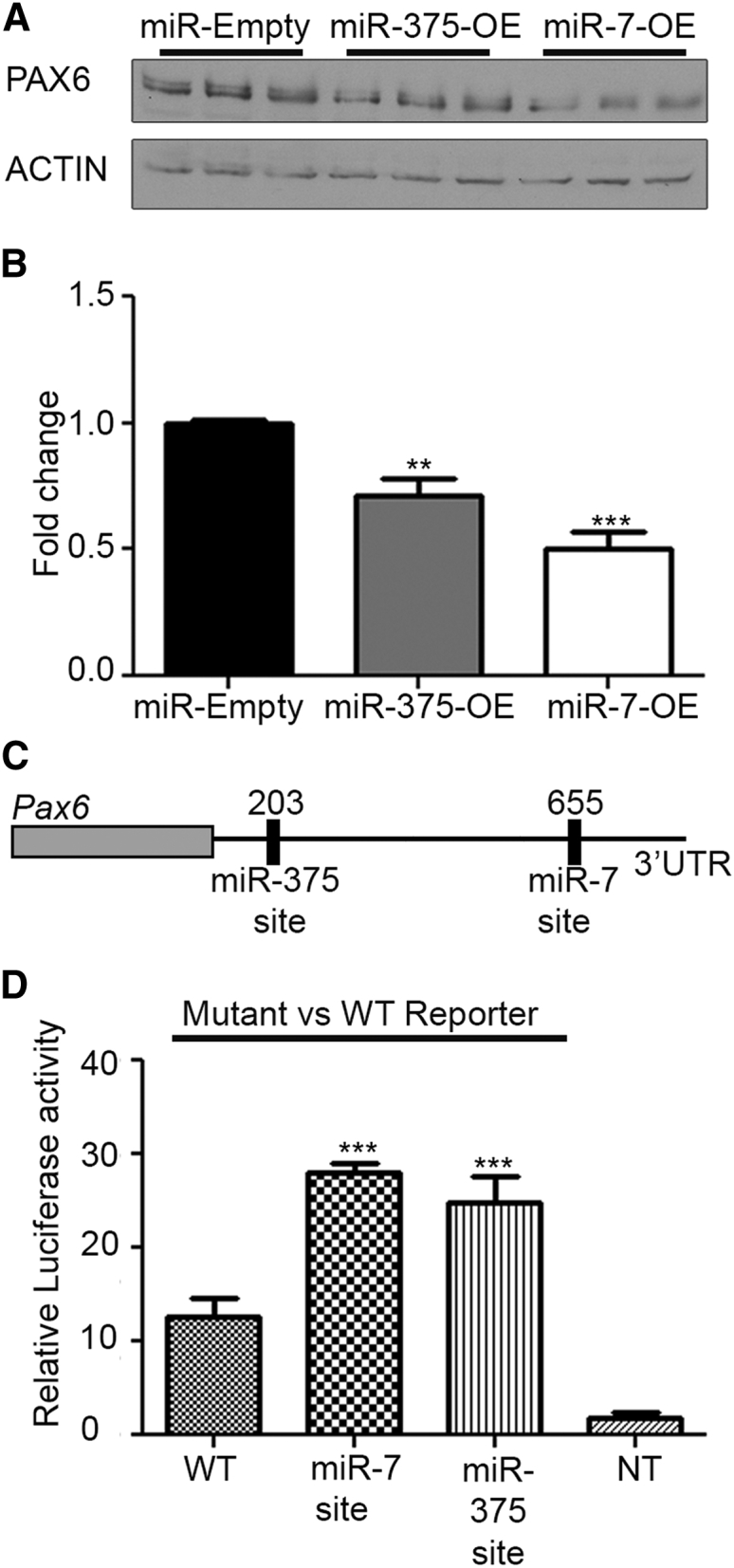
miRNA-375 and miRNA-7 Can Target *Pax6* 3′ UTR in β-TC-6 Cells (A) Representative western blot of the effect of miRNA-375 or miRNA-7 overexpression on PAX6 protein levels in β-TC-6 cells (three independent transfections); β-actin was used as a loading control. (B) Quantification western blot analysis; fold change is relative to miR-Empty. (C) Schematic diagram of miRNA-375 and miRNA-7 binding sites. The seed sites are located at 201–207 (miR-375) and 655–660 (miR-7-site). (D) A representative Luciferase reporter assay of β-TC-6 cells 24 hr after transfection with the wild-type *Pax6* 3′ UTR or mutated *Pax6* 3′ UTR reporters. NT represents non transfected cells. A one-way ANOVA followed by Dunnett’s multiple comparison test was used to determine whether there were significant differences between the groups and control. (B) (F[2,6]) = 61.96, p < 0.0001; (D) (F[5,12]) = 28.19, p < 0.0001); **p < 0.01; ***p < 0.001. Each experiment was repeated at least three times in triplicate. In each case, error bars represent SD.

*Pax6* is regulated by miR-375 and miR-7 in the brain and in cancer cell lines.^20^, ^25^ In mice, there is a single miR-375-5p (7-mer-A1) site at position 178–207 and a miR-7-5p seed site (7-mer-m8) at position 633–661.^26^ As a first step toward increasing *Pax6* expression in pancreatic cells, we sought to confirm whether or not these two miRNAs regulate *Pax6* expression in murine pancreatic cells. To this end, miR-7 or miR-375 were overexpressed in the β-TC-6 cell line and the endogenous PAX6 protein levels were examined. Overexpression of either miR-7 or miR-375 decreased PAX6 protein levels in cells to 50% or 70% of wild-type PAX6 levels respectively (Figures 1A and 1B). We next analyzed the miR-7 and miR-375 putative binding sites in the *Pax6* 3′ UTR to determine which sites were functional. We generated luciferase reporters in which the putative miRNA binding sites (Figures 1C and 1D) were mutated. Transient expression of each of the reporters in pancreatic cell lines, which endogenously express both miR-7 and miR-375, was performed, and the expression of the mutant reporters were compared to the wild-type *Pax6* 3′ UTR. The mutation of miR-7-site increased luciferase expression approximately 2-fold relative to the wild-type (WT) *Pax*6 3′ UTR reporter in β-TC-6 cells (Figure 1D) and confirms that this miR-7 site is important for miR-7 regulation of *Pax6* in these cells. The miR-375-site mutant reporter was also significantly increased relative to WT reporter (1.9-fold increase), indicating this site is also functional in pancreatic cells. Together, our data confirm that both miRNA-375 and miRNA-7 regulate *Pax6* expression in pancreatic cells.

### Tough Decoys against miRNA-7 and miRNA-375 Increase *Pax6* Reporter Expression

**Figure 2:**
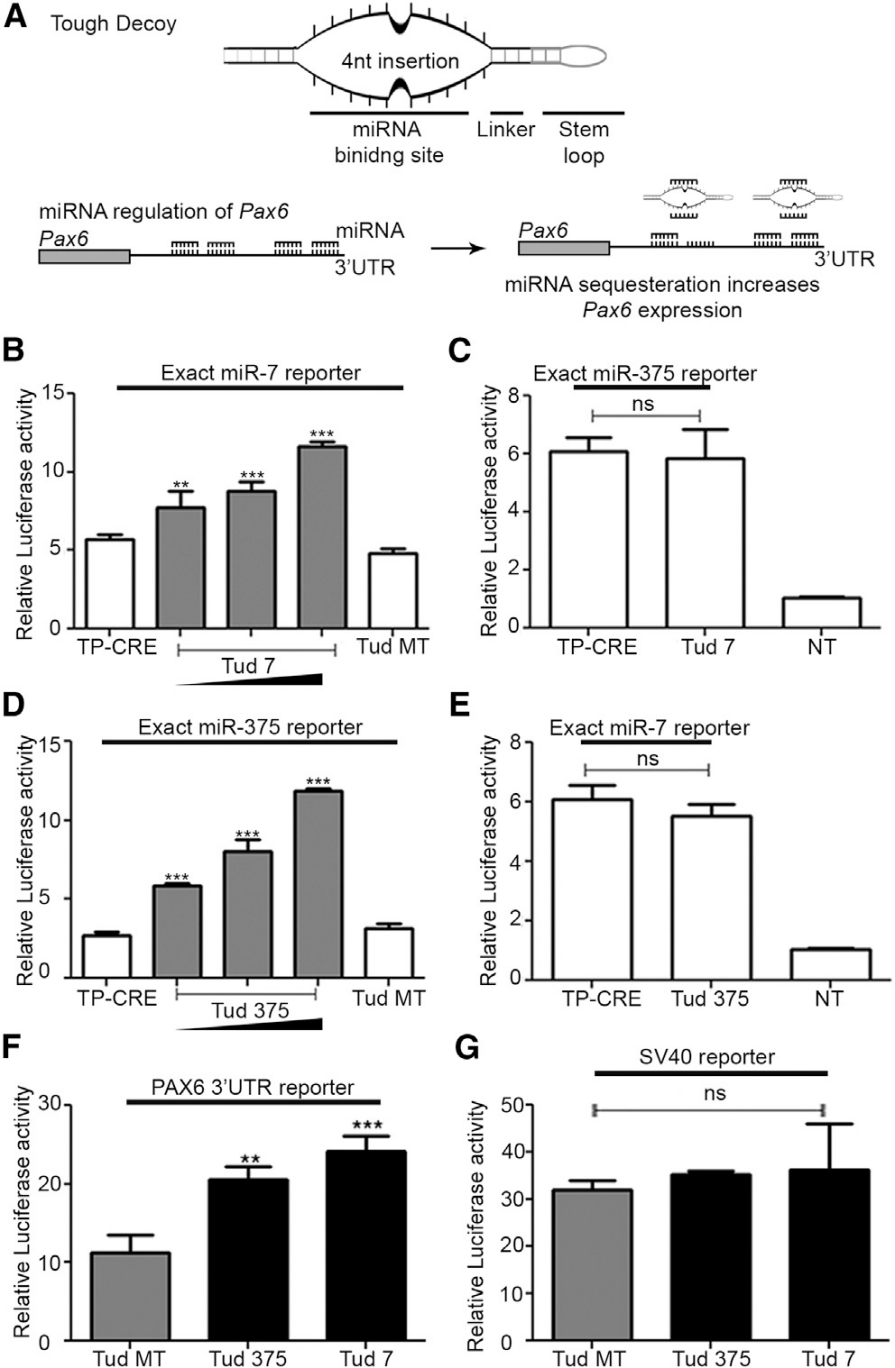
Tough Decoys Repress miR-7 and miR-375 (A) Schematic diagram of tough decoys (Tuds) designed against miRNA-375 and miRNA-7. The Tuds are designed to sequester miR-7 and miR-375, stopping miRNA-7 and miRNA-375 from interacting with their target seed sites. (B) A representative luciferase reporter assay of β-TC-6 cells 24 hr after co-transfection with the miR-7 seed reporter and either control TP-CRE, Tud MT, or increasing concentrations of Tud 7. (C) A representative luciferase reporter assay of β-TC-6 cells 24 hr after co-transfection with the miR-375 seed reporter and either control TP-CRE or Tud 7 (at the highest concentration in B). (D) A representative luciferase reporter assay of β-TC-6 cells 24 hr after co-transfection with the miR-375 seed reporter and either control TP-CRE, Tud MT, or increasing concentrations of Tud375. (E) A representative luciferase reporter assay of β-TC-6 cells 24 hr after co-transfection with the miR-7 seed reporter and either control TP-CRE or Tud 375 (at the highest concentration in D). (F) A representative luciferase reporter assay of β-TC-6 cells 24 hr after co-transfection of wild-type *Pax6* 3′ UTR and Tud constructs. (G) A representative luciferase reporter assay of β-TC-6 cells 24 hr after co-transfection with SV40 reporter and Tud constructs. For each set of data, a one-way ANOVA, followed by Dunnett’s multiple comparison test comparing all columns to the appropriate control, was used to determine whether there were significant differences between the groups and the control for each panel. (B) F[4,10] = 47.45, p < 0.0001; (C) F[2,6] = 61.10, p < 0.0001; (D) F[4,10] = 59.35, p < 0.0001; (E) F[2,6] = 28.19, p < 0.0001; (F) F[2,6] = 33.69, p < 0.0001; (G) F[2,6] = 0.4674, n.s.; **p < 0.01; ***p < 0.001; n.s., non-significant. Each experiment was repeated at least three times in triplicate. In each case, error bars represent SD.

We next determined the impact of inhibition of miR-375 or miR-7 on PAX6 protein levels. Utilizing the “tough decoy” (Tud) strategy developed by Haraguchi et al.,^27^ we first designed a miR-7 and miR-375 Tud (Figure 2A). Tuds function as miRNA sponges, blocking the action of their target miRNA.^27^ To validate our Tud 7 (Tud targeting miR-7) and Tud 375 (Tud targeting miR-375), we first transfected a luciferase reporter that contains a miR-7 seed site downstream of luciferase reporter into β-TC-6 cell lines. This reporter was co-transfected with either a control plasmid, which expressed RNA from bacterial CRE recombinase under the control of the H1 promoter (TP-CRE), a vector expressing Tud vector with the miRNA binding site mutated (Tud MT), or Tud 7 at increasing concentrations. Increasing concentration of Tud 7 increased the expression of the miR-7 site reporter in β-TC-6 cells, up to 2-fold relative to the TP-CRE or Tud MT (Figure 2B). In contrast, Tud 7 had no effect on a luciferase reporter that contains the miR-375 seed site (Figure 2C), confirming Tud 7 is specific for miR-7. Similarly, Tud 375 was able to increase the expression of the miR-375 site reporter in β-TC-6 cells (Figure 2D) but had no effect on the miR-7 site reporter (Figure 2E). These results indicate that the Tud 7 and Tud 375 are effective at specifically blocking the actions of their respective miRNAs.

**Figure 3:**
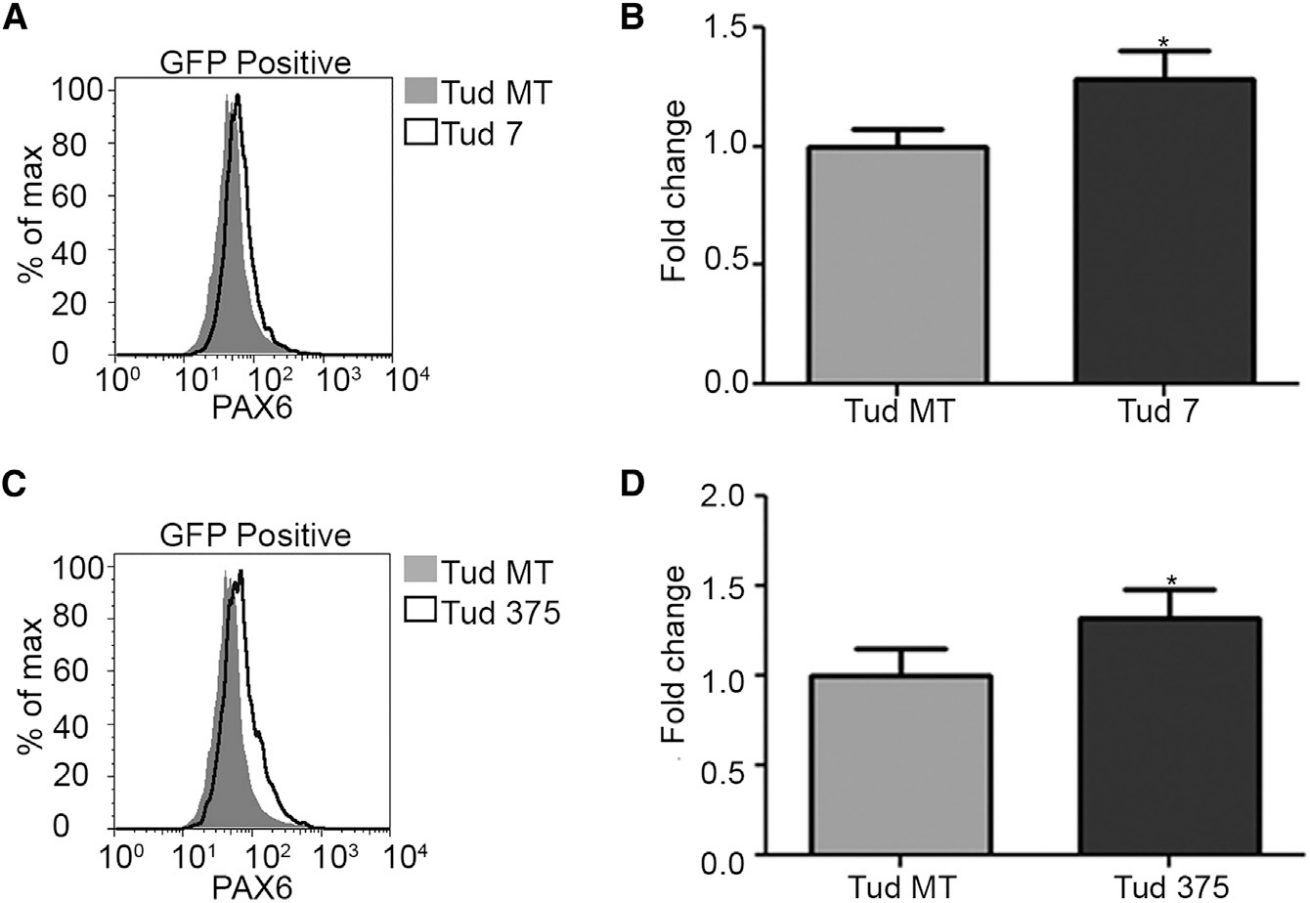
Tud-Mediated Inhibition of miRNA Increases PAX6 Protein Levels in β-TC-6 Cells (A) Histogram of PAX6 expression in β-TC-6 cells transfected with Tud MT (shaded) or Tud 7 (non-shaded). (B) Quantification of histogram for Tud MT and Tud 7 showing the fold-change in PAX6 expression relative to Tud MT. (C) Histogram of PAX6 expression in β-TC-6 cells transfected with Tud MT (shaded) or Tud 375 (non-shaded). (D) Quantification of histogram for Tud MT and Tud 375 showing the fold-change in PAX6 expression relative to Tud MT. A two-tailed t test was used to determine whether there was a significant difference between Tud MT and either Tud 7 (B) or Tud 375 (D); in (B), t (4) = 3.559, p = 0.0236; in (D), t (4) = 3.281, p = 0.0305. *p < 0.05. The fold change for both (B) and (D) is the average geometric means from three biological replicates normalized to the Tud MT. Each experiment was repeated at least three times in triplicate. In each case, error bars represent SD.

To examine the effects of inhibition of miR-7 and miR-375 on *Pax6* expression, we examined Tud-mediated miRNA inhibition on a luciferase reporter that contains the murine *Pax6* 3′ UTR immediately downstream of the luciferase cDNA. Similar to the exact seed site reporters, both Tud 7 and Tud 375 were able to specifically block the actions of miR-7 or miR-375 on the *Pax6* 3′ UTR increasing reporter expression in β-TC-6 cells by 2.2- or 1.8-fold, respectively, relative to Tud MT (Figure 2F). The effects on the reporter were specific, as neither Tud had any effect on an SV40 3′ UTR luciferase reporter (SV40 reporter), which does not contain seed sites for miR-7 or miR-375 (Figure 2G). Since our reporter assay measures luciferase enzymatic activity and not the protein level directly, we next determined the effect of Tud 7 or Tud 375 on endogenous PAX6 protein levels. β-TC-6 transfected with Tud 7 or Tud 375 constructs showed a modest increase in PAX6 levels of approximately 1.3-fold relative to Tud MT (Figures 3A–3D). Collectively, our data indicate that miR-7 and miR-375 regulate expression of *Pax6* in pancreatic β-TC-6 cells and blocking this regulation modestly increases PAX6 protein levels.

### Target Protectors against miR-7 or miR-375 Increase *Pax6* Expression

**Figure 4:**
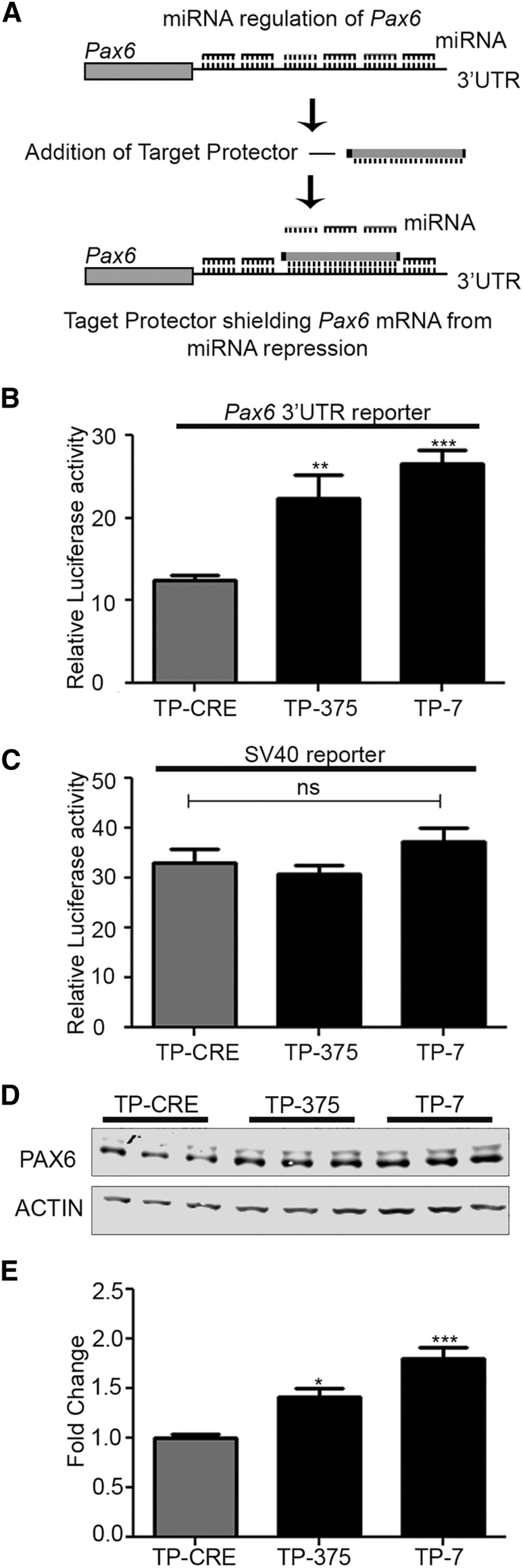
Target Protection of miRNA Seed Sites within the *Pax6* 3′ UTR Increases *Pax6* 3′ UTR Reporter Levels (A) Schematic diagram of target protector (TP) strategy. The TPs are designed to shield the miR-7 or miR-375 binding sites in the Pax6 3′ UTR, stopping miRNA-7 or miRNA-375 from interacting with their target seed sites. (B) A representative luciferase reporter assay of β-TC-6 cells 24 hr after co-transfection with wild-type *Pax6* 3′ UTR reporter and TP constructs. (C) A representative luciferase reporter assay of β-TC-6 cells 24 hr after co-transfection with SV40 reporter and TP constructs. (D) Representative western blot of three independent experiments of the effect of TP-375 or TP-7-mediated repression on PAX6 protein levels in β-TC-6 cells; β-actin was used as a loading control. (E) Quantification of western blot analysis, fold change relative to TP-CRE. For all panels, a one-way ANOVA followed by Dunnett’s multiple comparison test was used to determine whether there were significant differences between the groups and the control (TP-Cre). (B) F[2,6] = 43.00, p = 0.0003; (C) F[2,6] = 0.67276, n.s.; (E) F[2,6] = 23.17, p = 0.0015; **p < 0.01; ***p < 0.001; n.s., non-significant. Each experiment was repeated at least three times in triplicate. In each case, error bars represent SD.

Having established the suitability of targeting miR-7 and miR-375, we next sought a strategy to specifically limit the effects of this targeting to the *Pax6* 3′ UTR and further enhance the levels of *Pax6* expression. To this end, we designed target protectors to the *Pax6* 3′ UTR to specifically interfere with the miR-7 or the miR-375 site. Our target protectors (TPs) are 68-bp RNA molecules that specifically target, via complementarity, miRNA sites within target mRNA and prevent miRNA binding.^28^ However, because of their size, TPs may block binding of more than one miRNA site. In our case, our TP-7 is predicted to interfere with miR-7 as well as adjacent sites: miR-495, 96, 200b, 200c, 429, 876, 590-3p, 376c, 182, and 203 (B.C.R. and R.L.C., unpublished data). Similarly, our miR-375 TP (TP-375) is predicted to also block the adjacent miR-182, miR-365, miR-212, miR-369, and miR-374 sites. We reasoned that this may be advantageous and further boost PAX6 levels (Figure 4A). To control for non-specific effects, we used a TP complementary to the bacterial CRE recombinase cDNA (TP-CRE), which does not interact with any mammalian transcripts. Expression of either TP-375 or TP-7 increased the expression of the *Pax6* 3′ UTR luciferase reporter in β−TC-6 cells by 1.8- or 2.2-fold for TP-375 and TP-7, respectively, relative to the control (Figure 4B). Importantly, neither TP had any effect on the SV40 reporter showing that the effects of the TPs are specific to *Pax6* 3′ UTR (Figure 4C). We next sought to determine the effect of the TPs on PAX6 protein levels in these cells. Consistent with TP-7 and TP-375’s ability to target more than one miRNA, western blot analysis revealed that both TP-375 and TP-7 were able to increase PAX6 protein levels 1.4- or 1.8-fold in β-TC-6 cells, respectively (higher than achieved with Tuds; Figures 4D and 4E). These results demonstrate that the TP strategy is effective at boosting PAX6 protein levels within the 2-fold range we sought.

### AAV-2-TP-7 Increases PAX6 Protein in Islets

**Figure 5:**
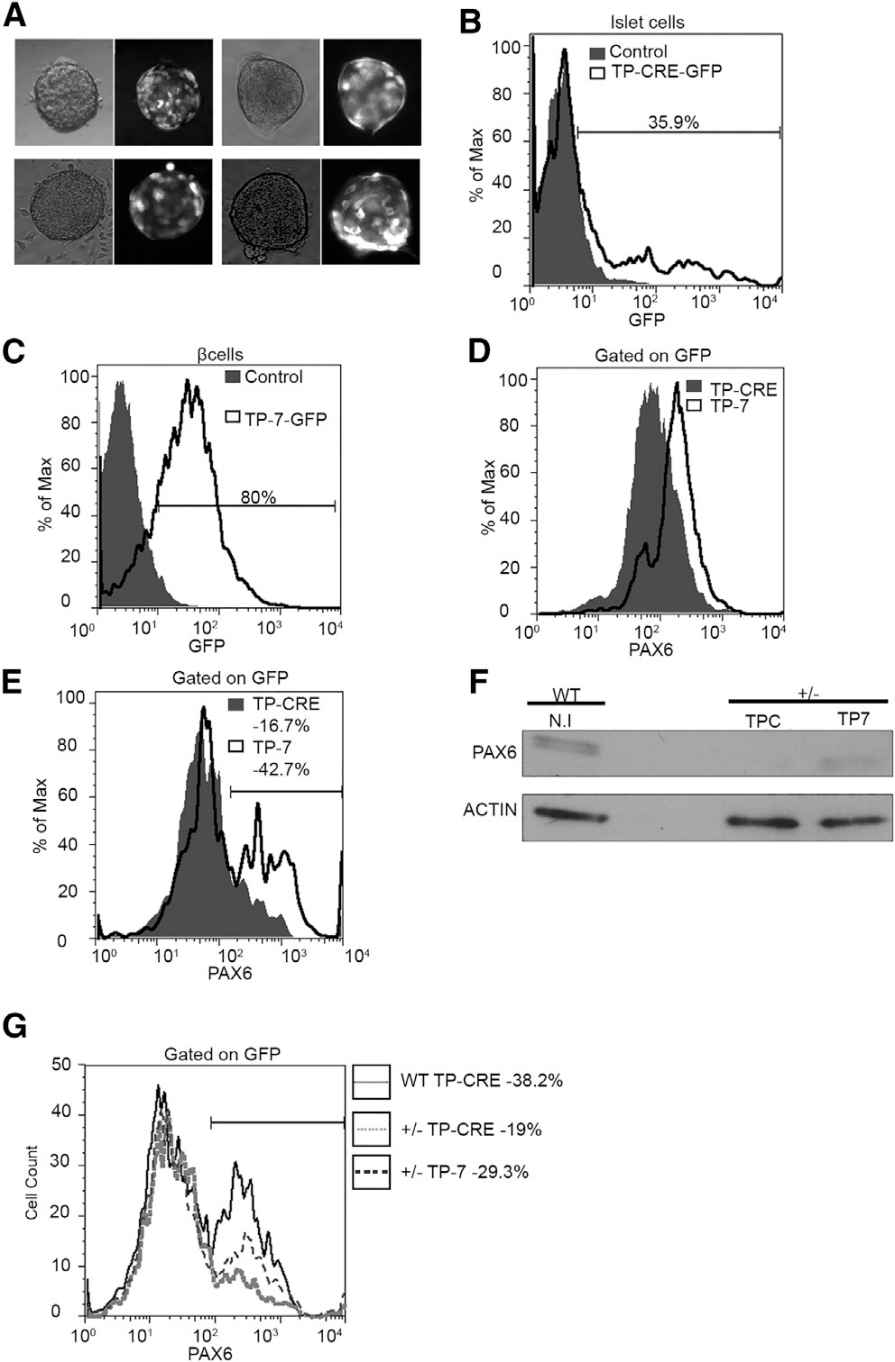
AAV-2 Mediated Delivery of TP-7 into Isolated Islets Results in Partial Recovery of PAX6 Protein Levels (A) Representative bright-field and fluorescence images of islets treated with AAV-2 expressing GFP 5 days post-transduction. (B) A representative GFP histogram of dissociated islet cells; the shaded histogram represents non-infected islet cells, while the non-shaded histogram with the dark line represents cells treated with AAV-2-TP-CRE-GFP. This experiment was repeated at least three times. (C) GFP histogram from β-TC-6 cells. This experiment was repeated at least three times. (D) PAX6 histogram from GFP-positive β-TC-6 cells. (E) PAX6 histogram from GFP-positive dissociated wild-type islet cells. This experiment was repeated at least three times and included blind replicates in which the treatment condition was unknown to the analyzer. In (D) and (E), the shaded histogram represents cells or islets treated with TP-CRE, and the non-shaded histogram with the dark line represents cells or islets treated with TP-7. (F) Western blot analysis of the effect of TPs on PAX6 protein levels in *Pax6*^*wt*^ and *Pax6*^*SeyDey*+/−^ islet cells either non-infected (NI) or treated with TP-Cre (TPC) or TP-7; β-actin was used as a loading control. This experiment was repeated twice. (G) PAX6 histogram from GFP-positive islet cells; the histogram with the solid black line represents dissociated wild-type islet cells treated with TP-CRE, the histogram with small dashed gray line represents dissociated islets from heterozygous mice treated with TP-CRE, and the histogram with large dashed line represents dissociated islets from heterozygous mice treated with TP-7. This experiment was repeated twice. In each case, error bars represent SD.

An important question is whether the TP strategy can be used to restore PAX6 levels in *Pax6* haploinsufficient cells. It is possible that in *Pax6*-deficient cells, miRNA regulation may already be adjusted to compensate for low PAX6 levels. In which case, interfering with miRNA regulation may be of no benefit. To address this question, we sought to restore *Pax6* expression in islets from mice heterozygous for the *Pax6*^*SeyDey*−/+^ mutation. This is a spontaneous deletion of the *Pax6* gene and heterozygous mutant mice have characteristic small eyes, which is accompanied by coloboma, lens defects, abnormal folding of the retina, reduced pigment layer, and an overall small body size. To target the TPs to islets, we used an adeno-associated virus (AAV-2), which contained a GFP cassette, in which GFP is driven by chicken β-actin (CAG) promotor, and a TP cassette, in which TP expression is controlled by the ribonucleic acid polymerase III (RNAP III)-dependent H1 promoter. AAV-2 was effective at targeting the islets *ex vivo* based on GFP fluorescence (Figures 5A and 5B). The targeting efficiency was approximately 35% of the islet cells (Figure 5B). To confirm the ability of AAV-2-TP-7 to target β cells, we transduced the β-TC-6 cells. Approximately 80% of β-TC-6 cells expressed the GFP reporter confirming the ability of AAV-2 to target this lineage (Figure 5C). We next gated for GFP and measured PAX6 expression in β-TC-6 cells using flow cytometry. The majority of the TP-7 AAV-targeted β-TC-6 cells overexpressed PAX6 relative to the TP-CRE control, confirming the ability AAV-2 delivered TP-7 to target the endogenous *Pax6* (Figure 5D). AAV-2-TP-7 infection of WT islets resulted in overexpression of PAX6 in 43% of the infected cells (Figure 5E). Based on these results, we next compared the effect of TP-7 on PAX6 levels between WT islets and islets isolated from *Pax6*^*SeyDey+/*−^ mice. Isolated islets from WT and heterozygous *Pax* ^*SeyDey+/*−^ mice were infected with either AAV-TP-7 or AAV-TP-CRE. After treatment of TP-7, heterozygous islets displayed an increase of PAX6 protein to ∼70% of WT levels using western blot analysis (Figure 5F). One difficulty with protein quantification from whole-cell lysates is that one cannot discern between increases in expression in the majority of cells versus overexpression in a small minority of cells. This is an important issue, since overexpression of PAX6 can have deleterious consequences. To address this issue, we performed flow cytometry on infected dissociated islet cells. As expected, PAX6 levels were reduced relative to WT in islets from heterozygous animals expressing the TP-Cre control virus. Expression of TP-7 in heterozygous islets restored expression of PAX6 to levels that were intermediate between the heterozygous TP-CRE and the WT TP-CRE islets and did not result in PAX6 overexpression (Figure 5G). These results show that *Pax6* haploinsufficient cells remain sensitive to the effects of miR-7 on *Pax6* expression and that TP-7 is effective at increasing PAX6 levels within physiological range.

## Discussion

In the present study, we evaluated whether the miRNA regulating *Pax6* can be therapeutically targeted to restore PAX6 protein levels in islets isolated from a mouse model of aniridia, called the small eye dickie (*Pax6*^*SeyDey*^) mouse. Our results provide evidence that TPs against the miR-7 site within the 3′ UTR of *Pax6* can partially restore PAX6 protein levels in pancreatic islets from small-eye mice bearing a heterozygous *Pax6* mutation.

*Pax6* is an essential regulator of pancreatic development and physiology.^29^, ^30^, ^31^, ^32^, ^33^ Several studies have shown that *Pax6* plays an important role in endocrine cell development and glucoregulation.^4^, ^29^, ^30^, ^31^, ^32^, ^33^, ^34^, ^35^, ^36^ For instance, pancreatic conditional knockout of *Pax6* in mice results in perinatal lethality and exhibit deficits in the maturation of hormone-producing lineages.^29^, ^37^ Mice with heterozygous *Pax6* mutations exhibit micropthalmia and develop age-dependent diabetes, which can be exacerbated by a high-fat diet.^37^, ^38^, ^39^ The diabetes is attributed to the role of *Pax6* in regulation of pancreatic hormone expression and processing in both the pancreas and gut, where *Pax6* transcriptionally regulates the expression of insulin, incretins, proconvertase 1/3, glut2, glucagon, and somatostatin.^7^, ^31^, ^40^, ^41^, ^42^, ^43^, ^44^ The requirement for human *PAX6* for glucoregulation has also been identified in aniridia patients who display age-dependent glucose intolerance, attenuated early insulin responses, and diabetes.^4^, ^33^, ^34^, ^45^ More recently, a non-coding allelic variant of *PAX6* in linkage disequilibrium with diabetes has been identified in Scandinavian populations and affects *PAX6* expression in human pancreatic islets.^45^ This suggests *PAX6* has a broader role in the pathophysiology of diabetes in some populations. Therefore, our results in targeting miRNA regulation of *PAX6* pancreatic expression have potentially broad implications for therapy of prediabetes and diabetes.

We first showed by miRNA overexpression, miR-7 and miR-375 reporters, *Pax6* 3′ UTR reporters, and Tud inhibitors of miR-7 and miR-375, that miR-7 and miR-375 regulate PAX6 protein levels within the β-TC-6 insulinoma cell line. Both miR-7 and miR-375 are key regulators of pancreatic physiology. Knockout of miR-7 in adult islet β cells *in vivo* leads to defects in insulin secretion and diabetes. Similarly, in a murine miR-375 knockout model, miR-375 has been shown to regulate glucose homeostasis and control α and β cell mass.^24^ In part, these effects reflect regulation by miR-375 on glucose-stimulated insulin secretion through myotrophin and its ability to control pyruvate dehydrogenase kinase 1 (PDK1) levels, a critical downstream effector of insulin signaling. Our results show that *Pax6* is also a target of miR-7 and miR-375 in pancreatic cells and it will be interesting to determine whether *Pax6* contributes to some of the effects of miR-375 and miR-7 on pancreatic physiology.

An important issue associated with *Pax6* gene therapy is that too much PAX6 can be just as detrimental as too little.^9^, ^11^, ^46^ Our data show that in mouse cells, blocking of miR-7 or miR-375 modestly raises PAX6 protein levels less than 2-fold. This makes miRNA regulation of *Pax6* an ideal target for therapeutic intervention. One concern that we had going into these studies was that in heterozygotes for *Pax6*, there may have been compensatory changes in miRNA regulation that would negate any effects of blocking miRNA in this context. Importantly, we found that a TP against the miR-7 within the 3′ UTR restored PAX6 expression in heterozygous islets to approximately 70% of WT. Therefore, these data indicate that *Pax6* mRNA remains under miRNA regulation in a heterozygous setting. Our TP 7 blocks the seed sites for several miRNA. Among these, miR-376c, 182, and 203 are also expressed in pancreatic α and β cell lines and physically interact with the *Pax6* 3′ UTR (B.C.R. and R.L.C., unpublished data). Thus, the effect of TP 7 in islets may not be solely ascribed to miR-7. However, given the abundance of miR-7 in pancreatic tissue, the strong effect observed in the pancreatic insulinoma cell line with Tud 7 and mutational analysis of the miR-7 site, it is likely miR-7 is a major contributor to the effects seen with TP 7 in pancreatic cells. Further optimization of TP length, including evaluating the use of peptide nucleic acids, could be used to further enhance specificity.^47^, ^48^, ^49^ However, our results show that derepression of the WT *Pax6* allele by a TP targeting the miR-7 site increased PAX6 expression without resulting in overexpression.

For the treatment of aniridia, it is desirable to have TPs for *Pax6* expression in the eye. The progressive vision loss in aniridia suggests that intervention may be possible to stop the progression and preserve vision. Encouragingly, Ataluren treatment of post-retinal development juvenile mice showed improvements to both the cornea and retina with topical application to the cornea.^50^, ^51^ Whether this will hold true in humans awaits the results of the current clinical trials (ClinicalTrials.gov: NCT02647359). *PAX6* expression in the cornea is required to maintain limbal stem cell populations, and its reduction in aniridia patients leads to corneal clouding and keratinitis.^52^, ^53^, ^54^, ^55^ Unfortunately, we currently do not know the miRNA that regulate *Pax6* in the cornea. However, miR-7 is expressed in the retina and may regulate *Pax6* expression here.^56^ Further work is required to determine the appropriate miRNA target sites within the *Pax6* 3′ UTR and eye structures to target.

A critical feature of haploinsufficiency disorders such as aniridia is that patients have a WT copy of the gene. By targeting the stable message from the WT allele, TPs are effective regardless of the type of mutation. An added advantage to TPs is that they only function in the cells where the gene is already expressed, which reduces the possibility of off-target effects in non-target tissues.^48^ This is in contrast to nonsense-mediated decay inhibition methods, such as Ataluren, which targets the nonsense mediated decay (NMD) pathway and mutant allele in the hope that increasing the concentration of mutant protein will improve function.^50^ Mutations resulting in premature stop codons are thought to represent approximately 50% of aniridia patients.^50^ In these patients, the mRNA from the mutant premature stop-containing allele is normally destroyed through NMD. A difficulty with targeting NMD is that it is difficult to predict ahead of time whether the mutant allele is functional, and there is a risk that some mutations may result in proteins that exacerbate the phenotype. However, TPs interfere with the miRNA-mRNA pairing within the 3′ UTR of the message from the WT allele.^28^, ^48^, ^49^ Perfect complementarity allows the TP to outcompete the miRNA binding to the seed site and therefore de-represses the miRNA regulation of the transcript. In principle, this approach could be applied to other haploinsufficiency disorders, including monogenetic diabetes (MODY) and neurofibromatosis type 1, or to upregulate homozygous mutants where the mutant protein retains some function, as was recently shown for cystic fibrosis transmembrane conductance regulator (CFTR).^47^

In conclusion, our data provide an important a proof of concept, demonstrating that the targeting miR-7 repression of *Pax6* using TPs can restore expression of PAX6 without resulting in overexpression in pancreatic tissue. Thus, our results provide impetus for *in vivo* investigation of the therapeutic value of TPs against the miR-7 binding site within the 3′ UTR of *Pax6* as a means of boosting PAX6 levels without undue overexpression. The important next questions are (1) whether can we target *Pax6 in vivo* in the pancreas and (2) whether increasing *Pax6* in adult mice can protect against age-related diabetes and high-fat diets in aniridia mouse models.

## Materials and Methods

### Animal Care Statement

This study was carried out under the guidelines of the Canadian Council on Animal Care. The protocol was approved by the University of Victoria Animal Care Committee (permit number: 2013-011(1-3)). All mice were group housed within HEPA-filtered ventilated racks within the animal care unit. All cages included enrichment in the form of huts, crinkle paper, nestlets, and sunflower seeds. Mice were subject to daily monitoring for adverse clinical signs. Euthanasia was carried out under isoflurane anesthesia. The *Pax6*^*SeyDey+/*−^ mice were obtained from Jackson Lab.

### Cell Culture

β-TC-6 (ATCC) were cultured in growth medium consisting of DMEM (Hyclone) supplemented with 15% heat-inactive bovine growth serum (BGS) and 1 mM sodium pyruvate. Cell lines were cultured at 37°C and 5% CO_2_. Mouse islets were cultured in RPMI media (Gibco) supplemented with 10% heat-inactivated BGS and 1% penicillin-streptomycin (Gibco). Islets were initially cultured at 27°C and 5% CO_2_ for 48 hr, before being incubated at 37°C and 5% CO_2_.

### Plasmid Sequence

The Luciferase reporters were based on cloning the appropriate sequences into the pMir-Luc (Signosis) reporter. Table 1 shows the nucleotides in *Pax6* 3′ UTR mRNA sequence found in WT *Pax6* 3′ UTR and the corresponding mutations. The WT *Pax6* 3′ UTR reporter contains the full-length mouse *Pax6* 3′ UTR (Gene ID, 18508) which is comprised of 2,055 base pairs. In addition, miR-375 and miR-7 sequences were cloned within the pMir-Luc 3′ UTR to create reporters sensitive to these miRNA. The sequences utilized for the miRNA reporters are shown in Table 1.

**Table 1:** *Pax6* 3′ UTR and miRNA Reporter Sequences

Name	Detail	Sequence (5′–3′)
*Pax6* 3′ UTR	186–207	GCACGGUAUCAGUUGGAACAAA
*Pax6* 3′ UTR miR 375 site	186–207 mutant	GCACGGUAUCAGUUGG**GG**CAAA
*Pax6* 3′ UTR	640–662	AAAAUGUAAGUAUUUGUCUUCCC
*Pax6* 3′ UTR miR7 site	640–662 mutant	AAAAUGUAAGUAUUUGUC**GG**CCC
miR-375 reporter	–	CTAGTTCACGCGAGCCGAAC**GAACAAA**A
miR-7 reporter	–	CTAGTAAAATGTAAGTATTT**GTCTTCC**A

Mutation sites are shown in bold. For the miR-375 and miR-7 reporters, the seed sites are shown in bold.

The Tuds and TPs were subcloned into plasmid containing adeno-associated virus genome (pAAV)-LacZ vector purchased from Agilent Technology. The TPs and Tuds were designed to be expressed from the Pol III-driven H1 promoter. To control for non-specific effects, we used a TP complementary to the bacterial CRE recombinase mRNA (TP-CRE), which should not interact with any mammalian transcripts. Blast searches were performed on all TPs to identify sequences unique to *Pax6*. The Tuds against miR-7 or miR 375 were developed according to the method described by Haraguchi et al.^27^ In brief, each Tud was designed to contain two miRNA binding sites complementary to the mature miRNA sequence, a 4-nt insert, a stem loop to aid in export to cytoplasm, and a 3-bp linker. Table 2 shows the Tud and TP sequences that were utilized.

**Table 2:** Sequences of Tuds and TPs Utilized

Name	Sequence (5′–3′)
Tud-MT	$GACGGCGCTAGGATCATC\underline{\text{AAC}}\text{AGGGGGGAATCAC}ATCT\text{TAGTCTTCC}\underline{\text{CAA}}\underline{\underline{\text{GTATTCTGGTCACAGAATAC}}}\underline{\text{AAC}}\text{GGGGGGAATCAC}ATCT\text{TAGTCTTCC}\underline{\text{CAA}}GATGATCCTAGCGCCGTC$
Tud-375	$GACGGCGCTAGGATCATC\underline{\text{AAC}}\text{TCACGCGAGCCG}ATCT\text{AACGAACAAA}\underline{\text{CAA}}\underline{\underline{\text{GTATTCTGGTCACAGAATAC}}}\underline{\text{AAC}}\text{TCACGCGAGCCG}ATCT\text{AACGAACAAA}\underline{\text{CAA}}GATGATCCTAGCGCCGTC$
Tud 7	$GACGGCGCTAGGATCATC\underline{\text{AAC}}\text{CAACAAAATCAC}ATCT\text{TAGTCTTCCA}\underline{\text{CAA}}\underline{\underline{\text{GTATTCTGGTCACAGAATAC}}}\underline{\text{AAC}}\text{CAACAAAATCAC}ATCT\text{TAGTCTTCCA}\underline{\text{CAA}}GATGATCCTAGCGCCGTC$
TP-Cre	GGGACCGATTTCGACCAGGTTCGTTCACTCATGGAAAATAGCGATCGCTGCCAGGAGCG
TP-375	TGAATAAAAGTTTGGATACCAAAATGAAGATTTGTTCCAACTGATACCGTGCCTTCTGTACGCAAAGG
TP-7	TATTATAGAAATCATTCTGAGGATTTCTAGGGAAGACAAATACTTACATTTTGACATAAAACAAATTG

The stems of the Tuds are illustrated in bold, the linker is underlined, and the miRNA binding sites are shown in black with the 4-nt inserts shown in italics. The stem loop sequence is double underlined.

### Islet Isolation

Islets were isolated following a modified version of the isolation protocol from Zmuda et al.^57^ In brief, mice were euthanized, and a laparotomy was performed. The entry into the duodenum was clamped using a haemostat. 2 mL Liberase TL (Roche)-RPMI serum-free mix was perfused through the common bile duct into the pancreas using a 27G needle. Once perfused, the pancreas was removed and placed on ice. Perfused pancreases were placed in 37°C water bath for 14–18 min to facilitate digestion by Liberase TL. Following incubation, tissue was disassociated by shaking the tubes vigorously 40 times for 10 s each pulse. The pancreas was then washed with G-solution (1× Hank’s balanced salt solution [HBSS] supplemented with 1% BSA and 0.357 g/L sodium bicarbonate) to quench digestion. The digested pancreas was spun down at 290 × *g* for 2 min at 4°C. After a second wash step, the dissociated tissue was passed through a 0.419-mm steel sieve (Canadawide Scientific) to separate out non-digested tissue, fat, and lymph nodes. After the third wash, cells were purified on a 1110 Histopaque (Sigma) gradient. The gradient is then overlaid carefully with RPMI media. The samples were spun at 900 × *g* for 20 min at 24°C, with slow acceleration and no braking. After, the spin islets appear as a white band in between the 1100 Histoplaque and RPMI overlay. The layer in the gradient containing the islet cells was collected using a disposable 10-mL serological pipette into a new 15-mL conical tube and washed in G-solution. Islets were then purified further by gravity sedimentation by resuspending in G-solution and left to sit on ice for 5 min. The top layers contained cells and debris, while the islets settled to the bottom of the tube. Islets were then handpicked using a dissecting microscope and pooled according to size.

### Western Blots

For western blots, cellular lysate was subjected to 10% SDS-PAGE and immunoblotted with mouse anti-PAX6 1:100 (Developmental Studies Hybridoma Bank) and mouse anti-beta-ACTIN 1:4,000 (Sigma-Aldrich). The secondary antibody was either anti-mouse immunoglobulin G (IgG) horseradish peroxidase (HRP) conjugate (R&D Systems) at a working dilution of 1:4,000 to 1: 10,000, or anti-mouse IgG (H&L) (goat) Antibody DyLight 680 Conjugated (Rockland) at a dilution of 1:10,000. Blots were detected using Li-Cor Odyssey CLX imaging system, and analysis was undertaken using Image Studio software. For western blots of islets, 100 islets collected from at least three different animals of the same genotype were used for each well. Data and statistical analysis was done using Graph Pad Prism 7.0.

### Flow Cytometry

Cells were dissociated using trypsin into single-cell suspension. For % GFP determination, cells were dissociated and immediately run on a BD FACS Calibur flow cytometer. ∼1.0 × 10^4^ cells (from cell lines) or ∼1.0-5.0 × 10^4^ cells (for dissociated islets) were analyzed per sample. For flow cytometry of islet preparations, 30 islets collected from at least three different animals of the same genotype were pooled and used for each sample. For immunofluorescence quantification, single-cell suspensions were fixed with 1% parafomaldehyde (PFA) in PBS for 15 min in the dark at 4°C. Following fixation, samples were spun (all spin steps are 350 × *g*, 10 min unless otherwise indicated), cells were resuspended with cold (−20°C) 70% ethanol added dropwise, and incubated in the dark at 4°C on a nutator for an hour. Samples were spun down, and supernatant was removed. Samples were permeabilized with 0.1% Triton X-100 for 10 min on ice and then spun down. The supernatant was discarded, and cells were then resuspended and blocked in 3% BSA in PBS for an hour on a nutator. Samples were spun down and supernatant was removed. Samples where then washed with PBTB (PBS, 0.5% BSA, 0.1% Triton X-100). Samples were spun down, and supernatant was removed. Samples were then resuspended in PBTB + 1% goat serum + anti-mouse PAX6 (Developmental Studies Hybridoma Bank) (1:3,000 or 1:100 for islets) and/or PBTB + 1% goat serum + anti-chicken GFP (Abcam) (1:200) and incubated overnight on a nutator in the dark at 4°C. Samples were then washed twice with 3% BSA in PBS. The cells were then re-suspended in PBTB + 1% goat serum with the secondary antibody: F(ab’)_2_ goat anti-mouse IgG (heavy and light chain [H+L]) secondary antibody, Alexa Fluor 647 conjugate (Thermo Fisher) (1:1,000), and/or Alexa Fluor 488 AffiniPure F(ab’)_2_ donkey anti-chicken IgY (IgG) (H+L) (Jackson Immunoresearch Laboratory) (1:100) and incubated in the dark at 4°C for 2 hr on a nutator. Samples were then washed with 3% BSA in PBS then re-suspended in 0.5% BSA in PBS; cells were passed through 100-μM cell strainer and ran on the flow cytometer. Events were acquired on a BD FACS Calibur flow cytometer and analyzed using FlowJo software.

### Luciferase Assay

Cells were transfected with firefly and renilla luciferase reporter as indicated using JetPrime transfection reagent according to the manufacturer’s instructions. 24 hr following transfection, Dual-Glo Luciferase assay was performed according to manufacturer’s instructions (Promega). Data and statistical analysis was done using GraphPad Prism 7.0 software.

### Viral Transduction

Media was aspirated and replaced with serum-free media. The cells were then transduced with AAV viruses at an MOI of 10^6^ genetic copies (GC) per cell for cell lines and an MOI of 10^10^ GC per islet. All AAV stocks were purchased from Vector Biolabs. Three days post-transduction for cell lines and five days post-transduction for islets, samples were processed.

## Author Contributions

Conceptualization, P.L.H., K.Y., R.L.C.; Methodology, P.L.H., S.C.A., K.Y.; Investigation, K.Y., B.C.R., S.C.A; Writing – Original Draft, P.L.H., K.Y.; Writing – Review and Editing, P.L.H., K.Y., R.L.C., B.C.R.; Funding Acquisition, P.L.H., R.L.C.; Resources, P.L.H; Supervision, P.L.H.; Project Administration, P.L.H.

## Conflicts of Interest

The authors have no conflicts of interest.
